# Highlights from the 5th Symposium on Biological Data Visualization: Part 2

**DOI:** 10.1186/1753-6561-9-S6-S1

**Published:** 2015-08-13

**Authors:** Jan Aerts, G Elisabeta Marai, Kay Nieselt, Cydney Nielsen, Marc Streit, Daniel Weiskopf

**Affiliations:** 1 Visual Data Analysis Lab, ESAT/STADIUS, KU Leuven, Kasteelpark Arenberg 10, 3001 Leuven, Belgium; 2 iMinds Medical IT, KU Leuven, Kasteelpark Arenberg 10, 3001 Leuven, Belgium; 3 Electronic Visualization Lab, University of Illinois at Chicago, 851 S. Morgan St, Chicago, IL 60607, USA; 4 Center for Bioinformatics, University of Tübingen, Sand 14, 72076 Tübingen, Germany; 5 Department of Pathology and Laboratory Medicine, University of British Columbia and British Columbia Cancer Agency, 675 West 10th Avenue, Vancouver, British Columbia, V5Z 1L3, Canada; 6 Institute of Computer Graphics, Johannes Kepler University Linz, Altenberger Straße 69, 4020, Linz, Austria; 7 VISUS, University of Stuttgart, Allmandring 19, 70569 Stuttgart, Germany

## 

The expanding experimental methodology and resulting data of biological research create significant challenges for computational visualization techniques. The goal of BioVis 2015 - *the 5th Symposium on Biological Data Visualization *- is to organize a premier international and interdisciplinary event for all aspects of visualization in biology. The BioVis 2015 symposium is affiliated with ISMB, the Intelligent Systems for Molecular Biology conference, as a Special Interest Group (SIG). BioVis 2015 took place in Dublin, Ireland, July 10-11 2015.

All papers were reviewed by reviewers from both the bioinformatics and visualization fields and were evaluated for improvements over state-of-the-art and for scientific soundness. The review process was organized in two review cycles. In the first review cycle, each paper was reviewed by three to four reviewers. In the second review cycle, the primary reviewers checked whether the required revisions for conditionally accepted papers were successfully included. Based on the reviewers' scores, reviews, and recommendations, the BioVis 2015 Paper and Publication Chairs and *BMC Proceedings* Section Editor together selected those that would be published in *BMC Proceedings*.

The papers from BioVis 2015 appear in two different proceedings: As "Proceedings of the 5th Symposium on Biological Data Visualization: Part 1" in a *BMC Bioinformatics *supplement (http://www.biomedcentral.com/bmcbioinformatics/supplements/16/S11)and as "Proceedings of the 5th Symposium on Biological Data Visualization: Part 2" in these *BMC Proceedings*. The highest quality papers were selected for the *BMC Bioinformatics *supplement. Those papers that feature high quality work but were not accepted for the *BMC Bioinformatics *supplement are published in *BMC Proceedings*. From the 21 papers submitted to BioVis 2015, 9 papers are published in the *BMC Bioinformatics *supplement and 5 papers are published in these *BMC Proceedings*.

The articles in these Proceedings cover an interesting spectrum of challenging problems in biological data visualization and their solutions. First, several papers address the issue of pathway visualization. Dang et al. make two contributions, presenting both a glyph/matrix-based visualization to investigate one-to-one relationships between proteins [1], and a method for inferring causality within pathways [2]. In addition, Paduano and Forbes [3] describe an approach for multi-level exploration of hierarchical pathways and pathway comparison. Second, Jung et al. [4] describe their software for investigating the relationships between miRNA and mRNA expression levels. Finally, Rumpf et al. [5] approach the issue of how to represent conformational changes in molecules from a flow-visualization standpoint.

We hope that the collection of papers in these Proceedings will educate, inspire, and engage visualization researchers in problems in biological data visualization, as well as bioinformatics and biology researchers in state-of-the-art visualization research.

## Competing interests

The authors declare that they have no competing interests.

## Notes

This article has been published as part of *BMC Proceedings *Volume 9 Supplement 6, 2015: Proceedings of the 5th Symposium on Biological Data Visualization: Part 2. The full contents of the supplement are available online at http://www.biomedcentral.com/bmcproc/supplements/9/S6.
